# Plasma proteomics implicates NOX-driven redox imbalance in degenerative cervical myelopathy: findings from the Australian MYelopathy Natural History Registry [AO Spine RECODE-DCM research priority number 5]

**DOI:** 10.1080/13510002.2026.2649669

**Published:** 2026-03-28

**Authors:** Nashwa Najib, Valerie C. Wasinger, Ryan O'Hare Doig, Muhammad Alsherbiny, Stone Sima, Ashish D. Diwan

**Affiliations:** a Department of Orthopaedic Surgery, Spine Labs & Spine Service, Discipline of Surgery, St. George Hospital, Sydney, Australia; b St. George & Sutherland Clinical Campus, School of Clinical Medicine, UNSW Medicine & Health, University of New South Wales (UNSW), Sydney, Australia; c Centre for Orthopaedic & Trauma Research | Spine Labs Adelaide, Adelaide Medical School, Faculty of Health and Medical Sciences, Adelaide University, Adelaide, Australia; d Spinal Unit, Royal Adelaide Hospital, Adelaide, Australia; e Bioanalytical Mass Spectrometry Facility, Mark Wainwright Analytical Centre, University of New South Wales (UNSW), Sydney, Australia; f Neil Sachse Centre for Spinal Cord Research, Lifelong Health Theme, South Australian Health and Medical Research Institute (SAHMRI), Adelaide, Australia; g Pharmacognosy Department, Faculty of Pharmacy, Cairo University, Egypt

**Keywords:** Degenerative cervical myelopathy, biomarkers, diagnosis, biological basis, proteomics, oxidative stress, glutathione signalling, cytokine signalling

## Abstract

**Objectives:**

To identify disease-relevant pathways and biomarkers in degenerative cervical myelopathy (DCM) patients from the MYelopathy Natural History Registry.

**Methods:**

Shotgun bottom-up proteomics (DCM *n* = 20; controls *n* = 20) was performed using LC–MS/MS in DDA mode. Peptides were eluted over 90 min on an in-house manufactured C18 column. Differential proteins were validated using parallel reaction monitoring (PRM) on the same instrument over a 60-min gradient. Bioinformatics was conducted in Skyline and ELISA using Ella™.

**Results:**

Discovery proteomics highlighted acute phase and cytokine signalling with STAT1/STAT3 involvement. Targeted assays showed higher IL-6 and IFN-γ in DCM, consistent with a pro-inflammatory state. PRM indicated upregulation of NADPH oxidase complex cytochrome b-245 α-chain (p22 phox or CYBA) and glutathione reductase, alongside downregulation of extracellular glutathione peroxidase, a pattern consistent with NOX-driven reactive oxygen species generation and impaired glutathione redox homeostasis. Together, this provides human plasma evidence of systemic redox imbalance in DCM and nominate a mechanistic framework linking cytokine signalling to oxidative stress via NOX activation and disrupted glutathione cycling.

**Conclusion:**

Findings support the feasibility of a plasma ‘liquid biopsy’ to augment diagnosis and monitoring. The modest cohort size and potential confounding by age and adiposity, absolute quantification, multivariate adjustment, and external validation are warranted to establish specificity and clinical utility.

## Graphical abstract

**Figure:**
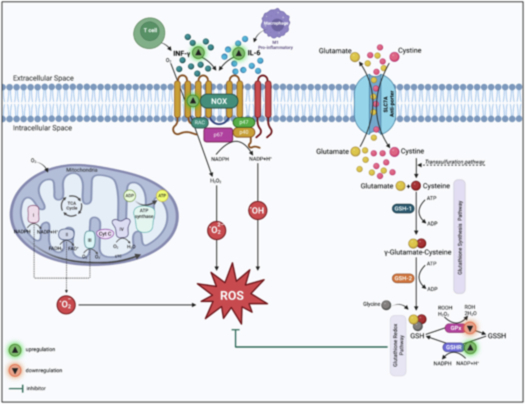

## Introduction

Degenerative cervical myelopathy (DCM) is a progressive degeneration of the cervical spine, leading to compression of the spinal cord [1,2]. It is characterised by progressive neurological symptoms such as pain in the neck, numbness, imbalance, bladder dysfunction and poor coordination [3]. Diagnosis relies on clinical examination and MRI evidence of degenerative changes in the cervical spinal cord and intervertebral discs. However, MRI poorly correlates with diverse clinical presentations and cannot detect underlying pathophysiological processes [4]. This gap underscores the need to identify the biological mechanisms that drive DCM. AO Spine RECODE-DCM [REsearch objectives and COmmon Data Elements for DCM] lists the biological basis as a research priority [5]. DCM pathophysiology has been studied in various animal models [6–8]. However, evidence directly linking systemic redox pathways to DCM in humans remains limited.

Oxidative stress is an imbalance between reactive oxygen species (ROS) and the body's protective mechanisms for their elimination. Oxidative stressors include infections, trauma, injury, surgery, and inflammation. NADPH oxidases (NOX) are a major source of intracellular ROS. Cytochrome b-245 is a component of NOX composed of two subunits: the alpha subunit (CYBA) and the beta subunit (CYBB/NOX2). In resting cells, NOX is dormant and activated by cytokines such as interferon gamma (IFN‑γ) and interleukin-6 (IL-6). NOX catalyses one-electron transfer from NADPH to O_2_, generating superoxide (O_2_•−) and downstream ROS. Glutathione (GSH) plays a crucial role in cellular defence against oxidative stress by neutralising free radicals and ROS [9]. Cystine enters the cell in exchange for glutamate through the SLC7A antiporter [10] to synthesise GSH via enzyme-driven pathways. The GSH/GSSG cycle, driven by glutathione peroxidase 3 (GPx3) and glutathione reductase (GSHR), buffers oxidative stress.

Plasma perfuses all body tissues, cells and circulation and is the major highway for both providing nutrients and removing cellular debris. Pathologies can readily be demonstrated by changes in the plasma proteome because of unique cellular signatures released into the bloodstream, making plasma an ideal ‘liquid biopsy’ that is readily accessible in the clinical setting. These findings may provide scope for early diagnosis and monitoring and improve insights into the biological basis of DCM [11]. Proteomics allows the study of protein interactions, function, composition, and structure, as well as their cellular activities [12]. Integrating global protein identification and pathway analysis to review the dysregulation of signalling networks and protein changes can enable a deeper understanding of DCM’s pathophysiological mechanisms.

The objectives of this study were (i) to identify differentially abundant proteins and pathways correlated with DCM and disease severity and/or gender and/or surgical status as a discovery phase and (ii) to establish a targeted protein panel using mass spectrometry to assess potential biomarkers of DCM.

## Methods

Twenty patients from the MYelopathy NAtural History (MYNAH) Registry [13] were included in this study. All participants provided written informed consent. This study was carried out in accordance with the principles of the Declaration of Helsinki (UNSW Ethics reference no.: iRECS3634). Plasma samples were obtained from the MYNAH Registry Biobank, collected only at baseline, at point of recruitment to MYNAH Registry. There were 11 males (55%) and 9 (45%) females with a mean age of 63 years and a mean BMI of 30 kg/m². Mild DCM was recorded in 14 (70%), moderate in 3 (15%) and severe in 3 (15%) using modified Japanese Orthopaedic Association (mJOA) score, and baseline surgical status (+) was recorded in 9 (45%) patients.

### Peptide isolation, protein assay and digestion

Albumin and IgG were depleted according to the manufacturer's notes using Thermo Scientific™ High Select™ Depletion Mini Spin Columns (Thermo Fisher Scientific, Australia, cat. no. A36365). The 2-D quant kit (Cytiva, via Sigma Aldrich Australia, cat. no. 80-6483-56) was used to determine protein amounts, and 100 μg of protein was enzymatically digested into peptides by adding trypsin (Promega, Australia, cat. no. V5111 or V511A) at a 1:100 enzyme-to-protein ratio in buffer containing 10 mM DTT, 2 M urea, and 50 mM ammonium bicarbonate at pH 8 overnight at room temperature and the reaction was stopped by acidifying the solution by adding 5 μL of neat formic acid (Optima LC‒MS grade, Thermo Fisher Scientific, Australia, cat. no. A117-50). The samples were desalted using C18 StageTips (Thermo Fisher Scientific, Australia, cat. no. 87784) [10]. Samples undergoing proteomic analysis were reconstituted in 10 μL of 0.1% formic acid.

### Global and parallel reaction monitoring proteomics

**Figure 1.**
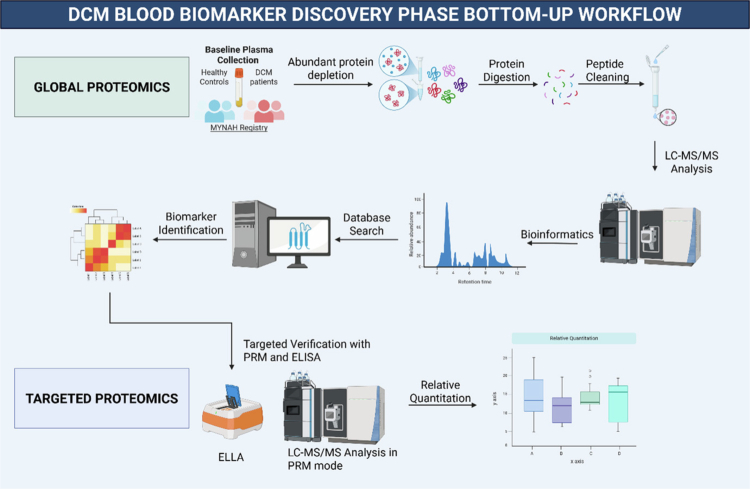
DCM blood biomarker discovery phase bottom-up workflow. DCM: degenerative cervical myelopathy, LC‒MS/MS: liquid chromatography‒tandem mass spectrometry, PRM: parallel reaction monitoring, ELISA: enzyme-linked immunosorbent assay.

Shotgun bottom-up proteomics (DCM = 20, healthy control = 20) was performed in Data Dependent Acquisition mode (Figure 1). One microgram of total protein was injected using LC–MS/MS on a Vanquish Neo Nano UPLC system and an Orbitrap Exploris 480 mass spectrometer (Thermo Fisher Scientific, Waltham, MA, USA) equipped with a high-field asymmetric waveform ion mobility spectrometer (FAIMS). Nanoelectrospraying was run in positive mode with a spray voltage of 2.1 kV and an ion tube temperature of 280 °C. FAIMS was operated at standard resolution with a nitrogen carrier gas flow of 4.6 L/min and a compensation voltage of −45 V.

All the peptides were eluted on an in-house manufactured 24 cm, 75 μm i.d., C18 column (1.9 μm, 120 Å, Dr. Maisch HPLC GmbH, Ammerbuch, Germany) using a linear gradient of H2O:CH3CN (98:2, 0.1% formic acid) to H_2_O:CH_3_CN (20:80, 0.1% formic acid) at 250 μL min^−1^ eluted over 90 min. Full MS scans were acquired in the Orbitrap at 45,000 resolution (m/z 350–1500) with an AGC target of 3.0 × 10^6^ ions and a maximum injection time of 50 ms. Dynamic exclusion was enabled (25 s duration, ±10 ppm mass tolerance) to minimise repeated precursor sequencing. Data-dependent MS/MS scans were triggered within a cycle time of 3.5 s between full scans. Precursors with charge states inclusive of 2–6 were selected with a 1.6 m/z isolation window using HCD fragmentation at 30% normalised collision energy. MS/MS were acquired in the Orbitrap at 15,000 resolution, with an AGC target of 2 × 10^5^ ions. Lock mass correction using EASY-IC™ was applied throughout the run.

PRM was achieved using FAIMS-enabled workflow on the same instrument with the same parameters. The PRM method consisted of a 60 min gradient optimised for an expected peak width of 23 s. Scans were acquired in the Orbitrap at 30,000 resolution using a 1.5 m/z isolation window and a normalised HCD collision energy of 30%–32%. The target peptides were monitored based on a predefined inclusion list. A secondary full MS scan was included for quality control at 30,000 resolution (350–1750 m/z), with FAIMS CV set to −45 V. Unique peptide transitions were developed in the Skyline [14] environment using the software's default settings. Data files were imported for manual inspection and interference assessment. The peak area under the curve of the parent ion was used to assess the relative abundance of the targets. PRM was used to analyse all product ions resulting from specific precursor ions [10]. The PRM of the unique signature parent and transition ions of the marker proteins (injection was 0.7 μl from 10 μl) are provided in Table 1, and a complete transition list is provided in Supplementary Table 2. The total ion current for each sample (DCM = 20, healthy control = 10) was used to normalise using relative quantitative techniques. A strict elution window of ±2 min and at least 3 transitions were used, when possible, to minimise false identifications.

**Table 1.** Fold change of the peptides that explain the redox homoeostasis and mitochondrial health in DCM.

Protein	Peptide	Accession Numbers	m/z	Fold change
NOX	K.LLGSALALAR.A	P04839	492.8137++	120.14
GSHR	R.LNAIYQNNLTK.S	P00390	646.3539++	33
GPx3	K.FLVGPDGIPIMR.W	P22352	657.8656++	0.03

Differentially expressed peptides identified by mass spectrometry. Peptide sequences, UniProt accession numbers, mass-to-charge ratio (m/z), and fold change between groups are shown. NOX = nicotinamide adenine dinucleotide phosphate oxidase; GSHR = glutathione reductase; GPx3 = glutathione peroxidase 3.

### Enzyme-linked immunosorbent assay (ELISA)

ELISA was conducted using Ella™ (ProteinSimple, Bio-Techne; instrument supplied in Australia by In Vitro Technologies, Brisbane, Australia). CRP and Cystatin-C (R&D Systems/Bio-Techne, via In Vitro Technologies, cat. nos. SPCKB-PS-000026 for CRP and SPCKB-PS-007569 for Cystatin-C) were diluted using a 2000-fold dilution, and IL-1b, IL-12 p70, IL-10, IL-2, IFN‑γ 3rd gen, TNF-α 2nd gen, IL-4 2nd gen, and IL-6 2nd gen (R&D Systems/Bio-Techne) using a 2-fold dilution. Diluted samples (DCM = 20, healthy control = 12) were loaded onto the respective ELLA cartridges and set to run for 90 min.

### Bioinformatics and statistical analysis

Proteins were identified using Mascot Daemon v2.5.1 (Matrix Science, London, UK) and searched against the SwissProt database (downloaded January 2023, containing 479,930 sequences). The search parameters were set to carbamidomethyl (C); variable modifications, oxidation (M), phospho (STY); enzyme, semi-trypsin; and maximum missed cleavages, 2; peptide tolerance, ±5 ppm; and fragment tolerance, 0.05 Da. Proteome Discoverer software (Thermo Fisher Scientific; Waltham, Massachusetts, US, v 2.4) was used to compare the proteome. Protein identifications were accepted with a ≤1% false discovery rate and contained at least 2 identified peptides. The expression changes across the samples were measured via the area under peak and normalised to the total ion count. ANOVA was used to report abundance changes controlled by the Benjamini‒Hochberg procedure for multiple comparisons correction, with adjusted *p*-values set to ≤0.05. The studies reached a power ≥90% and were calculated using PASS software based on mean abundance values and standard deviation between groups.

The proteomic dataset of differentially abundant proteins (fold change) was assessed for enriched pathways and causal networks using Ingenuity Pathway Analysis (IPA® Qiagen, Redwood City, CA, USA). The core analysis was carried out with only direct relationships and experimentally observed confidence considered, based on the IPA knowledge base (genes only). The *p*-value for the correlation between identified proteins and a given canonical pathway was calculated by Fisher's exact test; while a z-score of ≥2 or ≤–2 denoted higher or lower levels of pathway activation or inhibition, respectively.

Targeted protein data were analysed using Skyline software, and peptides were accepted based on retention time and sequence with at least 3 transitions when possible. The peak area under the curve of the parent ion was used to assess the relative abundance of the marker panel. Fold changes were calculated, and the data were evaluated using the Welch two-sample *t*-test, which was controlled by the Benjamini‒Hochberg procedure for multiple comparisons, with adjusted *p*-values set to ≤0.05. Analysis was conducted in R version 4.2.2.

## Results

**Figure 2.**
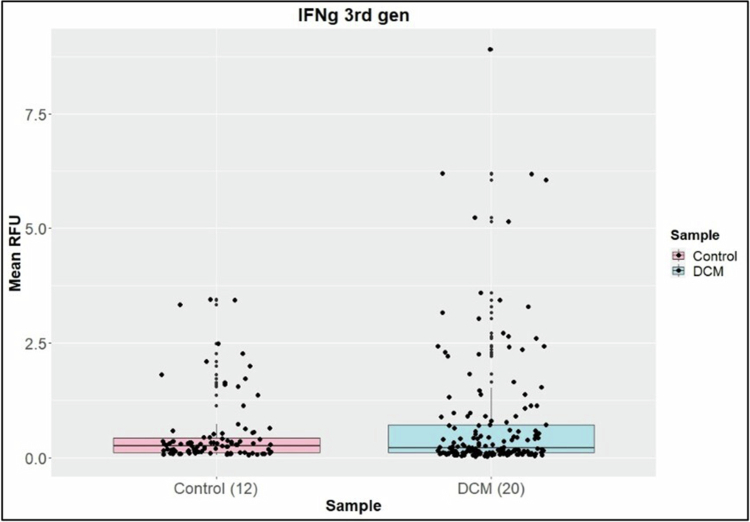
A comparison of the mean IFN‑γ (*p* = 0.05) RFU levels between the healthy control and DCM groups indicated upregulation in DCM patients.

**Figure 3.**
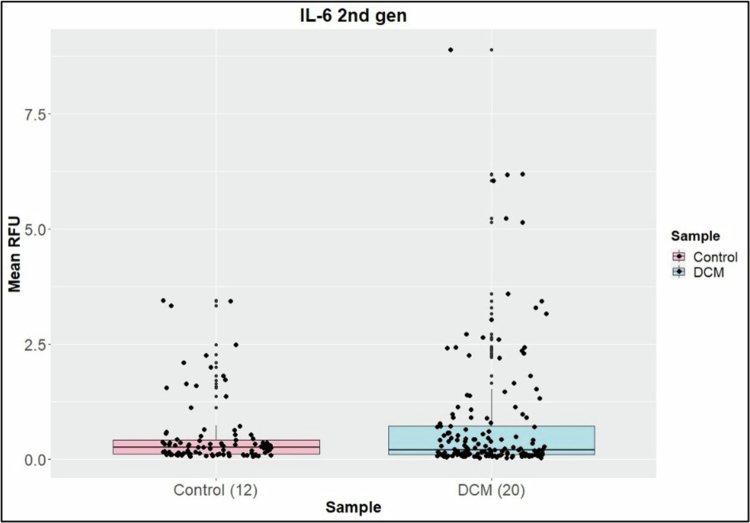
A comparison of mean IL-6 (*p* = 0.017) RFU levels between the healthy control and DCM groups indicated upregulation in DCM patients.

The DCM and control groups showed high similarity in protein numbers identified between sample groupings. Differential protein abundance ratios of DCM over Control were assessed. Using Fisher's exact test *p*-value (*p*< 0.05) and a score cut-off of −log (1.3) and a *z*-score > |1.8| showed that the list of 160 differential proteins had ties to immune function, ROS, cellular leak and membrane dynamics. A thorough study of the function of the proteins identified in the IPA was undertaken. No differences were found in CRP and Cystatin C between the surgical and non-surgical groups, gender, or DCM severity. No differences were found in IL-1b, IL-12 p70, IL-10, IL-2, TNF-α and IL-4 between DCM patients and healthy controls. IFN-γ (*p-*value = 0.05) (Figure 2) and IL-6 were found to be upregulated in DCM patients compared to healthy controls (*p-*value = 0.017) (Figure 3). Further subgroup analyses within the DCM patient group suggested that surgery status, DCM severity or gender did not influence the RFU levels of IL-1b, IL-12 p70, IL-10, IL-2, TNF-α 2nd-generation or IL-4. We targeted the CYBB complex and found that its two components—CY24B (*p*-value < 0.05) and NOX2 (*p*-value < 0.05)—upregulated in DCM patients compared to healthy controls.

**Figure 4.**
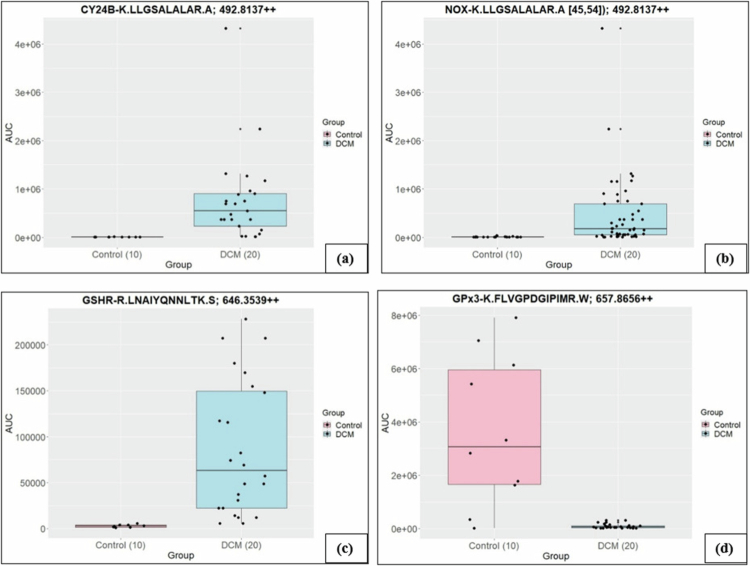
AUC values of peptides from PRM Proteomics. An upregulation of (a) CY24B (*p* < 0.05), (b) NOX (*p* < 0.05), and (c) GSHR (*p* < 0.05) in DCM patients was found; however, (d) GPx3 (*p* < 0.05) was downregulated.

The differential pathways between DCM and control patients can be distilled down to an over-representation in DCM with IFN‑γ involvement, resulting in granulocyte activation and resulting in connective tissue damage through degranulation and protease release. This inflammatory cascade is exacerbated by APOE involvement and the phagocytosis of cellular debris. The perpetuation of this cascade creates a positive feedback loop in which the outcome is increased ROS. Excessive ROS place a heavy burden on antioxidant systems; thus, we further explored the role of GSH as the primary intracellular redox buffer to compensate for immune-mediated inflammation and the management of APOE-related lipid peroxidation. The GSHR (*p*-value < 0.05) was also found to be upregulated in DCM patients compared to healthy controls. GPx3 (*p*-value < 0.05) was found to be downregulated in DCM patients compared to healthy controls (Figure 4).

### IPA-integrated summary and biological insights

**Figure 5.**
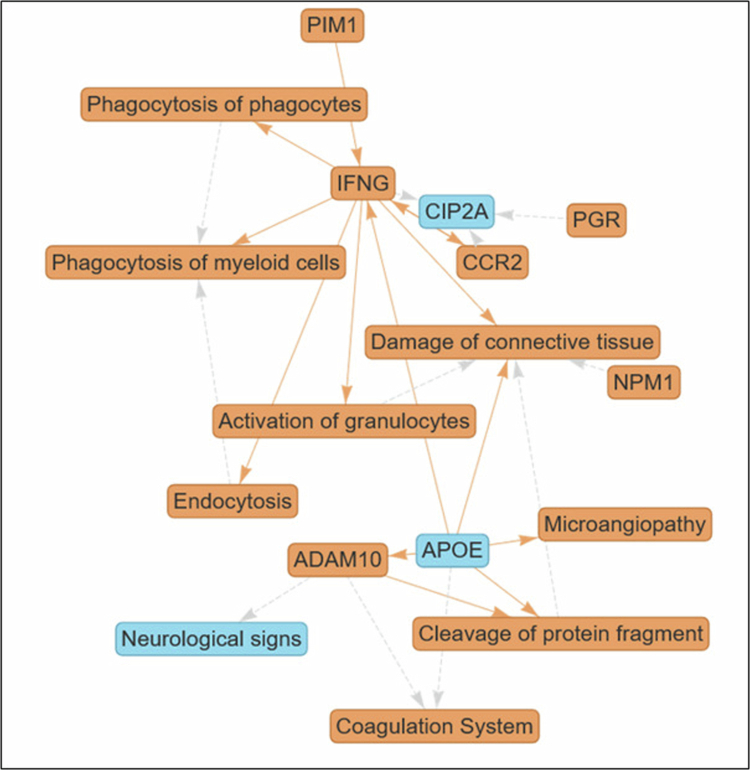
Granulocyte activation drives connective tissue injury. Distillation of the enriched pathways analysed by IPA Interpret in the form of a causal network with additional large language model (LLM)-driven summary highlights that the activation of granulocytes is triggered by IFN-γ to contribute to connective tissue damage.

Distillation of the enriched pathways analysed by IPA Interpret in the form of a causal network with an additional large language model (LLM)-driven summary highlights that the activation of granulocytes is triggered by IFN‑γ to contribute to connective tissue damage (Figure 5). These findings suggest REDOX homoeostasis involvement.

The network illustrates that an increase in IFN‑γ stimulates the activation of granulocytes, which in turn leads directly to increased damage of connective tissue. This cascade demonstrates an inflammatory process in which increased granulocyte function—potentially through degranulation and the release of proteases—disrupts tissue integrity, a hallmark of immune-mediated pathologies such as autoimmune disease or tissue injury in chronic inflammation.

ADAM10, a disintegrin and metalloproteinase, is positioned as a central regulator. Its increase promotes the cleavage of protein fragments and activates the coagulation system while also mitigating neurological signs. Given that a decrease in APOE leads to increased ADAM10, the network suggests a scenario in which loss of APOE function exacerbates proteolytic activity and coagulation, implicating this axis in neurovascular disorders or inflammatory conditions with vascular involvement. Decreased APOE expression is linked to increased levels of ADAM10, cleavage of protein fragments, coagulation system activity, IFNG, damage to connective tissue, and microangiopathy. This pattern identifies APOE as a key suppressor in this network, whose deficiency leads to a pro-inflammatory, pro-thrombotic phenotype. Such interactions are relevant in cardiovascular and neurodegenerative diseases where APOE function is compromised.

IFN‑γ is an upstream driver in this network, promoting granulocyte activation, expression of CCR2, endocytosis, phagocytosis (by both myeloid cells and phagocytes), and subsequent tissue damage. This highlights the role of IFN‑γ in amplifying macrophage and granulocyte responses, contributing to both pathogen clearance and collateral tissue injury, which are characteristic of hyperactive inflammatory states.

CIP2A, an endogenous inhibitor, is downregulated by increases in CCR2, IFN‑γ, and PGR. Since CIP2A acts as a brake on pro-inflammatory signalling, its reduction by multiple upstream signals suggests its role as a convergence point for intensifying inflammatory cascades. This makes it a potential regulatory bottleneck in the context of chronic inflammation or cancer-associated inflammation.

## Discussion

The acute phase response signalling pathway was the most enriched in the global proteomic analysis. This pathway is activated in response to infection, inflammation, trauma, surgery, or tissue injury and is mediated by cytokine signalling. An upregulation of pro-inflammatory cytokines IL-6 and IFN-γ in DCM patients suggested that chronic inflammation is at play. Increased IL-6 concentrations have been detected in CSF [15], while elevated serum IL-6 levels have also been reported in patients with degenerative disc disease [16], further underscoring its role in DCM pathology and neuroinflammation. IL-6 has been found to predict early postsurgical functional outcomes in DCM patients [17]. However, IL-6 is not DCM-specific; elevated levels of IL-6 are found in obese patients, and it has been found that adipose tissues are the main source of IL-6 [18]. There is also strong evidence that IL-6 levels increase with age [19]. The increased BMI and age of this cohort may explain the elevated levels of IL-6. IL-6 and IFN-γ play a key role in the activation of NOX. CY24B and NOX, both components of the NADPH oxidase complex, are implicated in oxidative tissue damage when dysregulated. NOX elevation has been consistently observed in neurodegenerative diseases [20], including Alzheimer's disease, Parkinson's disease, multiple sclerosis, and amyotrophic lateral sclerosis, which are conditions that share pathophysiological features with DCM, such as mitochondrial dysfunction and neuroinflammation.

In neurodegenerative diseases such as multiple sclerosis [21] and Parkinson's disease [22], elevated IFN-γ levels contribute to neuronal damage, glial activation, and disruption of the blood‒brain barrier—mechanisms that are also implicated in DCM pathophysiology. In the context of DCM, chronic spinal cord compression leads to immune cell infiltration and activation, resulting in sustained inflammation and oxidative stress. IFN-γ may exacerbate this process by promoting microglial activation and neuroinflammatory signalling, thereby contributing to axonal degeneration and functional decline. Its presence in plasma could serve as a biomarker of neuroinflammation in DCM, complementing other markers such as IL-6.

**Figure 6.**
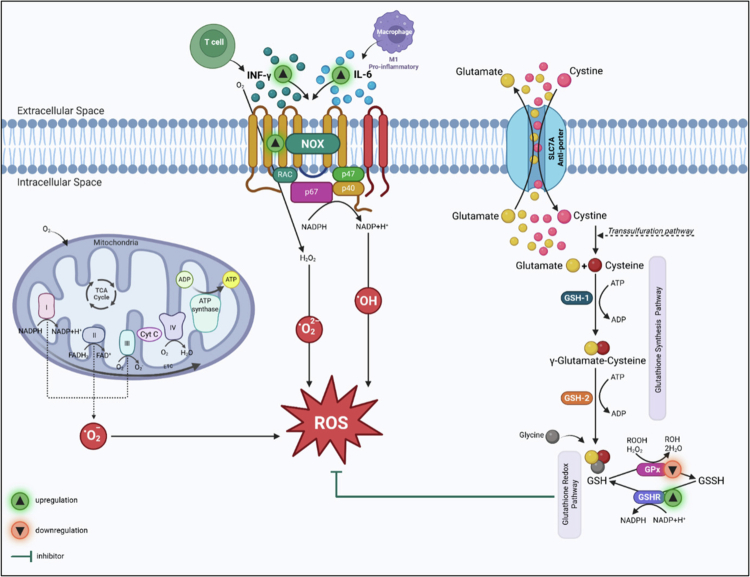
Biological pathways in degenerative cervical myelopathy (DCM).

The observed downregulation of GPx3 aligns with its established role in mitigating oxidative stress and inflammation [23–26] The impaired glutathione redox balance is the hallmark of oxidative stress [27], and most recently, the impaired GPx/GSHR axis has been found to reflect oxidative stress [10]. Oxidative stress has been observed in pre-clinical [28] and human studies [29], further supporting our findings (Figure 6).

### Strengths and limitations

By leveraging proteomics, the study achieved high-resolution profiling of protein expression, enabling the identification of protein signatures that may underpin DCM mechanisms. The integration of AI-bioinformatics tools further provided a layer of biological insight for robust interpretation of complex datasets. This research demonstrated translational potential, positioning proteomics as a powerful tool for bridging bench-to-bedside research. However, certain limitations warrant consideration. Symptom duration was not recorded, as the heterogeneity of DCM presentation and tracking patients to their first GP presentation/other allied healthcare practitioner for symptom complaint was not feasible. The relatively small sample size may limit the generalisability of the findings, including the lack of differences in biomarker levels across DCM severity groups. Additionally, proteomic data are inherently subject to variability owing to differences in sample preparation, instrument sensitivity, and data processing pipelines. Preexisting comorbidities, concurrent medications, meal timing, and the timing of sample collection may influence proteomic profiles. These factors can introduce variability and confounding effects. While efforts have been made to standardise procedures, some degree of technical bias may persist. Finally, these findings require further validation through absolute quantitation using internal heavy labelled standards for normalisation and standard curve generation to confirm their relevance and applicability in clinical settings.

## Conclusion

This study highlights the NOX-driven redox imbalance in DCM, encapsulating the triad of oxidative stress, inflammation, and neurodegeneration, which are the hallmarks of DCM pathophysiology. The findings support the feasibility of a quantitative blood test for diagnosis and monitoring.

## Ethics number

Ethical approval was obtained from the University of New South Wales (UNSW) Human Research Ethics Committee (HREC) Executive (ethics number iRECS3634), Australia.

## Consent

All participants provided written informed consent for this study.

## Supplementary Material

Supplementary material Supplementary Material

## Data Availability

Data can be requested via email sent to Dr. Nashwa Najib at nashwa.najib@adelaide.edu.au.
