# Supplementary material for: Plasma proteomics implicates NOX-driven redox imbalance in degenerative cervical myelopathy: findings from the Australian MYelopathy Natural History Registry [AO Spine RECODE-DCM research priority number 5]
Source: Redox Rep. 2026 Mar 28;31(1):2649669. doi: 10.1080/13510002.2026.2649669 (PMC13034710; doi:10.1080/13510002.2026.2649669)
Supplement: Supplementary material [file YRER_A_2649669_SM3393.docx]

***Supplementary material***

***MYNAH Registry Biobank–Blood collection, transport, and storage protocol***

The MYNAH Registry established a contract with Douglass Hanly Moir (DHM) Pathology Centres across Australia for the collection of blood. Blood (20 mL) was withdrawn using a 23G needle into 2 x 4mL EDTA tubes and 1 x 8.5 mL plain tube by a professional certified phlebotomist at DHM, adhering to their standard procedures. The EDTA tube was centrifuged at 1,500g for 15 minutes, and 750 μL plasma was transferred into 2 aliquot tubes, labelled as per DHM protocol, and frozen at -80 °C. The serum separator tube was allowed to clot for 30-60 minutes and kept on wet ice after clotting. The SST tube was centrifuged at 1,500g for 15 minutes, and 750 μL serum was transferred into 2 aliquot tubes, labelled as per DHM protocol, and frozen at -80 °C. Plasma and serum tubes were transported to the MYNAH Registry’s Biobank on dry ice, which, upon arrival, were de-identified, and 250 µL of plasma and serum were aliquoted in three cryotubes and stored at -80°C freezers until further use. Plasma samples of 20 healthy controls (age- and gender-matched) were acquired from the Australian Red Cross. All biospecimens were logged on the UNSW Open Specimen BioRegister (MySample).

***Explicit rationale for single draw blood at baseline***

Although ethical approval and participant signed consent permitted blood sampling at each follow-up visit, the study adopted a feasibility-oriented design to optimise resource utilisation and minimise participant burden. A single blood draw at baseline was deemed sufficient to address the objective of Discovery Proteomics, which requires only one high-quality sample per participant for comprehensive biomarker profiling. This approach reduced logistical complexity, costs, and potential attrition associated with repeated invasive procedures, thereby supporting participant retention and overall study integrity. The ongoing nature of the Registry ensures that additional samples can be obtained during future follow-up visits, enabling subsequent validation studies with a larger sample size for future studies.

***DCM evaluation using MRI***

MRIs of the patients were assessed, and the most recent MRI and radiology reports were used if multiple sets of scans were available for the same patient. Axial and Sagittal T1W and T2W MRI scans of the cervical spine were assessed. MRI data points were collected before reading the radiological reports. The presence, location, and association with T2W MRI of increased intramedullary signal intensity (IISI) were recorded. MRI degenerative parameters measured included nuclear degeneration (ND) (distinction between nucleus pulposus and annulus fibrosis), disc bulge (DB), endplate changes (EC), foraminal stenosis (FC), maximum spinal cord compression (MSCC), cervical tilt, and cranial tilt. The measurement protocols for IISI and radiological cervical degeneration parameters are detailed elsewhere. ^1^

***Radiological Presentation***

Out of 20 patients, 14 (70%) demonstrated increased intramedullary signal intensity (IISI) on MRI (Table 1). Among these, 11 patients had a single level of IISI, while 3 had two levels involved. All patients (100%) exhibited nucleus degeneration and disc bulge at least one cervical level, resulting in cord compression. Endplate changes were observed in 10 patients (50%), and foraminal stenosis was present in 17 patients (85%).

Radiographic measurements revealed an average cervical tilt of 8.56 ± 8.88 degrees and a cranial tilt of 13.04 ± 4.88 degrees. The most common levels of worst maximum spinal cord compression were C3–C4 and C5–C6, each in 7 patients (35%). The mean value of maximum spinal cord compression at the most compressed level was 0.759 ± 0.121.

This highlights the heterogeneity of MRI findings in DCM and underscores the importance of aligning radiological features with liquid biopsy.

Supplementary Table 1. Degenerative Intervertebral Disk and Spinal Cord Changes Observed in the DCM Cohort

| Parameter | IISI (n=20) | ND  (n=20) | DB  (n=20) | EC  (n=20) | FS  (n=20) |
| --- | --- | --- | --- | --- | --- |
| No. of patients (n, %) | 14 (70%) | 20 (100%) | 20 (100%) | 10 (50%) | 17 (85%) |
| C2-C3 (n, %) | 1 (5%) | 3 (15%) | 2 (10%) | 0 | 0 |
| C3-C4 (n, %) | 3 (15%) | 9 (45%) | 13 (65%) | 2 (10%) | 11 (55%) |
| C4-C5 (n, %) | 1 (5%) | 14 (70%) | 10 (50%) | 5 (25%) | 10 (50%) |
| C5-C6 (n, %) | 7 (35%) | 14 (70%) | 12 (60%) | 7 (35%) | 12 (60%) |
| C6-C7 (n, %) | 4 (20%) | 12 (60%) | 12 (60%) | 3 (15%) | 9 (45%) |
| C7-T1 (n, %) | 1 (5%) | 6 (30%) | 7 (35%) | 0 | 2 (10%) |

*Distribution of T2W MRI degenerative parameters across cervical levels. IISI= Increased intramedullary signal intensity, ND= nuclear degeneration (loss of distinction between nucleus pulposus and annulus fibrosis), DB = disc bulge, EC = endplate changes, FS = foraminal stenosis, MSCC = maximum spinal cord compression. Values are presented as number of patients (n) and percentage (%).*

Supplementary Table 2. Parallel Reaction Monitoring (PRM) Proteomics Transition List

| **Compound group** | **Protein name** | **Peptide sequence** | **Precursor Ion Mass** (m/z) | **Product Ion** (m/z) | **Colour** | **RT** (min) | **PPM** | **Chromatogram peaks** |
| --- | --- | --- | --- | --- | --- | --- | --- | --- |
| p\|P22352\|GPX3_HUMAN | Glutathione peroxidase 3 | K.QEPGENSEILPTLK.Y | 777.9041++ | G [y11] - 1200.6470+ | Yellow | 32.7 | 2.2 |  |
|  |  |  |  | N [y9] - 1014.5830+ | Yellow | 32.7 | 2.2 |  |
|  |  |  |  | S [y8] - 900.5401+ | Yellow | 32.7 | 2.2 |  |
|  |  |  |  | P [y4] - 458.2973+ | Yellow | 32.7 | 2.2 |  |
|  |  |  |  | E [b2] - 258.1084+ | Yellow | 32.7 | 2.2 |  |
|  |  |  |  | E [y7] - 813.5080+ | Yellow | 32.8 | 0.7 |  |
|  |  | K.FLVGPDGIPIMR.W | 657.8656++ | V [y10] - 1054.5714+ | Maroon | 40.2 | 0.3 |  |
|  |  |  |  | P [y8] - 898.4815+ | Maroon | 40.2 | 0.3 |  |
|  |  |  |  | D [y7] - 801.4287+ | Maroon | 40.2 | 0.3 |  |
|  |  |  |  | G [y6] - 686.4018+ | Maroon | 40.2 | 0.3 |  |
|  |  |  |  | P [y4] - 516.2963+ | Maroon | 40.2 | 0.3 |  |
|  |  |  |  | P [y8] - 449.7444++ | Maroon | 40.2 | 0.3 |  |
|  |  |  |  | G [b4] - 417.2496+ | Maroon | 40.2 | 0.3 |  |
|  |  |  |  | P [y4] - 258.6518++ | Maroon | 40.3 | 0.1 |  |
|  |  |  |  | L [y11] - 1167.6554+ | Maroon | 40.3 | 0.1 |  |
| sp\|P00390\|GSHR_HUMAN | Glutathione reductase | R.LNAIYQNNLTK.S | 646.3539++ | L [b9] - 1044.5473+ | Red | 28.4 | 23.4 | 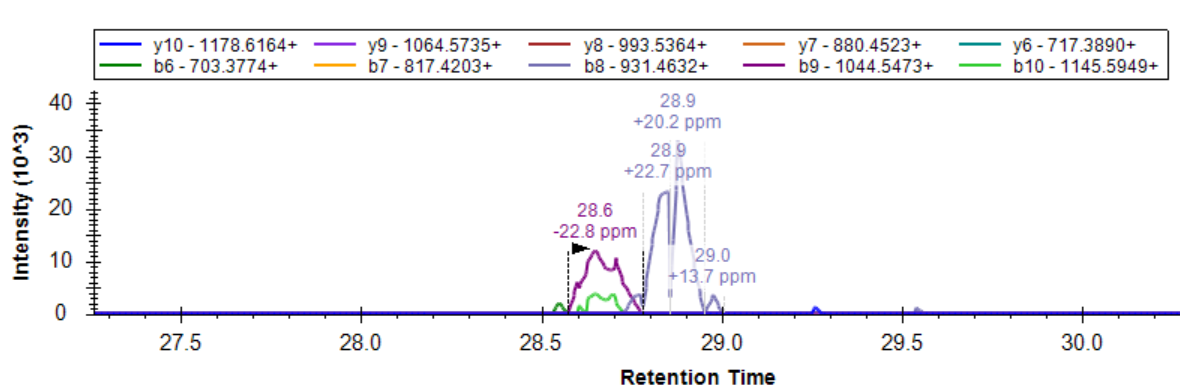 |
|  |  |  |  | T [b10] - 1145.5949+ | Purple | 28.4 | 23.4 |  |
| sp\|P04839\|CY24B_HUMAN | Cytochrome b-245 | K.LLGSALALAR.A | 492.8137++ | L [y9] - 871.5360+ | Purple | 26.3 | 4.1 | 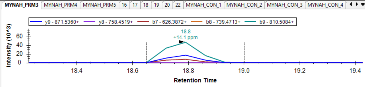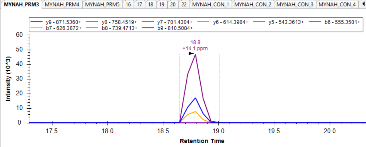 |
|  |  |  |  | A [b7] - 626.3872+ | Purple | 26.3 | 4.1 |  |
|  |  |  | 607.3179++ | V [y8] - 953.5455+ | Mustard | 16 | 20.7 |  |
|  |  |  |  | I [b7] - 821.3862+ | Mustard | 16 | 20.7 |  |
| sp\|P04839\|CY24B_HUMAN | NADPH Oxidase (NOX)  aka: Cytochrome b-245 heavy chain | K.LLGSALALAR.A [45, 54] | 492.8137++ | [y8] - 758.4519+ | Purple | 18.8 | 14.1 |  |

**References:**

1. Sial AW, Sima S, Chen X, et al. Spinal column radiological factors associated with increased spinal cord intramedullary signal intensity &#x2212; A study evaluating aging spinal cord&#x2019;s relation to spinal disc degeneration. *Journal of Clinical Neuroscience.* 2024;126:86-94.
